# Anti‐ageing activities of nanovesicles derived from *Artemisia* princeps in human dermal cells and human skin model

**DOI:** 10.1002/jex2.70033

**Published:** 2025-04-24

**Authors:** Kimin Kim, Yehjoo Sohn, Ju Hun Yeon

**Affiliations:** ^1^ Department of Integrative Biosciences University of Brain Education Cheonan Republic of Korea; ^2^ Well‐aging Exobio Inc. Cheonan Republic of Korea

**Keywords:** Anti‐aging, *Artemisia princeps*, MMP‐1, Nanovesicles, Procollagen type I

## Abstract

Plant‐derived substances are widely used as cosmeceutical and food materials owing to their beneficial properties that promote human health, such as antioxidant, nutritional supply and regenerative potential. In particular, nanovesicles (NVs) from plants contain various biomolecules, including signal proteins, nucleic acids, and metabolites, that participate in cross‐kingdom communication. In this study, we isolated NVs from *Artemisia princeps* (APNVs) based on differential centrifugation and further purification via tangential flow filtration (TFF). Evaluation of the effects of these NVs on the cellular proliferation of fibroblasts clearly indicated their anti‐ageing potential for the skin. Specifically, exposure of human dermal fibroblast cells to low concentrations of APNVs (100–200 ng/mL) accelerated cell proliferation over a 7‐day period. Treatment with APNVs decreased the senescence level of dermal fibroblast cells, as evidenced by senescence‐associated β‐galactosidase activity connected with cellular ageing. In the anti‐ageing efficacy assessment, inhibition of MMP‐1 activity in nanovesicle‐treated cells was higher than that induced by the positive control epigallocatechin‐3‐gallate (EGCG). To validate the inhibitory effect of APNVs on anti‐ageing in human skin, three‐dimensional, reconstituted human keratinocytes and dermal fibroblasts were cultured with 1000 ng/mL APNVs. Notably, procollagen type I expression was increased in the culture medium following APNVs treatment. Our collective results suggest that APNVs accelerate type I procollagen production through inhibition of MMP‐1. In view of the significant anti‐ageing potential of APNVs, we recommend their implementation as an active substance in pharmaceutical and functional cosmeceutical products.

## 1 INTRODUCTION

Plant‐derived nanovesicles (PDVs) possess intrinsic therapeutic activities that can be effectively employed to treat human diseases. These vesicles play an important role in intercellular communication and transportation of several bioactive molecules (Suharta et al., 2021), including DNA, RNA, proteins and other metabolites that function as cellular signals (Lian et al., 2022; Zhang et al., 2022). By facilitating intercellular delivery of bioactive molecules, extracellular vesicles (EVs) serve as cellular messengers that regulate physiological processes in multicellular organisms (Lian et al., 2022). The main attributes of PDVs include anticancer, anti‐inflammatory, antioxidant, and regenerative properties (Kim, Jung et al., 2020; Kim, Yoo et al., 2020; Nemati et al., 2022; Zhang et al., 2022). These NVs can successfully mediate intercellular communication between bioactive components owing to their small EV sizes (Shinge et al., 2022), providing a basis for their further development for multiple potential applications in the cosmetic and food industries (Cho et al., 2022; Suharta et al., 2021; Wang, Zheng, et al., 2023).

The genus *Artemisia* possessing anticancer, antioxidant, antidiabetic, anti‐inflammatory, and anti‐melanogenic properties has been historically used in traditional medicine (Bora & Sharma, 2011; Forouhandeh et al., 2023; Wang, Wei, et al., 2023). In a previous study, our group developed a technology to isolate plant‐derived EVs and reported the anti‐melanogenic effects of NVs from *Dendropanax morbifera* (Lee et al., 2020). However, at present, little is known about the properties of NVs of *Artemisia princeps* (APNVs).

In this study, we investigated the effects of APNVs on a skin ageing model. Analysis of physicochemical properties confirmed that these APNVs are nanoscale in dimension and non‐cytotoxic. To evaluate anti‐ageing effects, β‐galactosidase staining, matrix metalloproteinase‐1 (MMP‐1) and type I procollagen levels were examined in human dermal fibroblasts (HDFs) in addition to histological changes in a 3D skin model.

The APNVs (nanovesicles of *Artemisia princeps*) investigated in this study exhibited sizes comparable to those of naturally occurring animal cell exosomes and were predominantly spherical. NVs derived from human tonsil mesenchymal stem cells (TMSC‐NVs) demonstrated a significant anti‐ageing effect in a concentration‐dependent manner, as confirmed by the SA‐β‐galactosidase assay (Cai et al., 2020; Tan et al., 2016). APNVs effectively inhibited MMP‐1 secretion while enhancing type I procollagen secretion in TNF‐α and UV‐stimulated HDFs. Additionally, treatment with APNVs significantly restored the epidermal thickness of 3D‐cultured artificial skin treated with TNF‐α and increased collagen content in UVB‐irradiated human skin, as evidenced by Masson's trichrome staining.

These findings highlight the potential of plant‐derived NVs as new plant‐based materials for industrial applications, including cosmetics, food, and pharmaceutical areas. Currently, plant‐derived exosome cosmetics are being developed around the world, and the efficacy and absorption capacity of exosomes, which have plant‐specific effects, are attracting attention. In the food field, exosomes from various medicinal plants can be used to not only utilize the unique efficacy of each plant but also to safely consume them. n terms of pharmaceuticals, as the effectiveness of plant‐derived exosomes as a therapeutic agent is further verified and developed, it can become a safe and groundbreaking therapeutic candidate. If the plant‐derived exosome isolation process can be scaled up and mass‐produced, it can provide various strategies for commercial application.

## 2 MATERIALS AND METHODS

### 2.1 Isolation of A. princeps leaf‐derived NVs

Fresh *A. princeps* leaves were purchased from Misan Herb Farm (Daegu, Korea). The raw leaves were first juiced without solvent using an extractor and centrifuged at 5000 × *g* for 10 min to remove large debris. The filtered NVs were then concentrated and purified in a tangential flow filtration (TFF) system through a 500 kDa MWCO mPES hollow fibre MidiKros filter module using a peristaltic pump, further centrifuged at 5000 × *g* for 10 min at 4°C, and concentrated in an Amicon Ultra‐4 PL 100 K concentrator (Merck Millipore, Damstadt, Germany) (Lee et al., 2020). Finally, the supernatant was filtered through a 0.22 µm membrane to isolate APNVs. The NVs protein content was determined using the Pierce bicinchoninic acid (BCA) protein assay kit (Thermo Fisher Scientific, Waltham, MA, USA). Absorbance was measured at 562 nm using a microplate reader (BioTek, Winooski, VT, USA). All experiments in this study used only APNVs, which are NVs isolated from *A. princeps* leaf extracts.

### 2.2 Characterization of APNVs

To evaluate the hydrodynamic size distribution profiles of the different formulations, a Zetasizer nano ZS90 system (Malvern Panalytical, Malvern, UK) was employed. The scattered intensity autocorrelation function was used to acquire measurements of collected APNVs, which were placed in a thermostatic cell at 20°C. APNVs were mixed with distilled water at a ratio of 95:3 (v/v) for assessment of zeta potential. Each diluted sample was placed into a folded capillary cell (DTS1070; Malvern Instruments) and examined with the Zetasizer nano ZS90 system.

### 2.3 Transmission electron microscopy (TEM)

For TEM, a 4 µL sample solution containing APNVs was placed onto a Cu 200‐mesh carbon film grid that had undergone surface treatment with glow discharge (Electron Microscopy Science, PA, USA). An aliquot of solution (100 µL) containing 2% (w/v) uranyl acetate was used to stain the grid after incubation with the sample for 1 min and three washes with 20 µL distilled water. Grids were air‐dried for 10 min and viewed under a JEM‐2100F (JEOL Ltd, Tokyo, Japan) microscope outfitted with a field emission gun and OneView camera (Gatan Inc., CA, USA). Extra stain solution was removed using Whatman filter paper (GE Healthcare Life Science, Buckinghamshire, UK). An acceleration voltage of 200 kV accelerating voltage was applied.

### 2.4 Cell culture

HDFs, (CC‐2512; Lonza, Basel, Switzerland) were grown in fibroblast growth medium‐2 (FGM‐2; Lonza) on culture dishes under a 5% CO_2_ humidified atmosphere at 37°C.

### 2.5 Cytotoxicity and cell proliferation assays

For the cell viability assay, HDFs (2 × 10^4^ cells/well) were seeded into individual wells of a 96‐well plate and incubated for 24 h under 37°C and 5% CO_2_. Cells in each well were treated with a range of doses of APNVs (0, 100, 200, 500, and 1000 ng/mL).

For the cell proliferation assay, HDFs (2 × 10^4^ cells/well) were seeded in 96‐well plates, incubated for 24 h at 37°C and 5% CO_2_, and treated with APNVs at doses of 0, 100, 200, 500, and 1000 ng/mL for 1, 3, 6, 9, 12, 15, 18, and 21 days (*n* = 3).

For assessment of cytotoxic effects, plates were further incubated with EZ‐Cytox reagent (Daeil Lab Service, Seoul, Korea) and cell viability in response to APNVs was examined. Absorbance was measured at 450 nm using a microplate reader (BioTek, Winooski, VT, USA).

### 2.6 Senescence‐associated β‐galactosidase staining

Cellular senescence was visibly detected with the aid of senescence‐associated beta‐galactosidase (SA‐Gal) staining. To this end, HDFs were washed with phosphate‐buffered saline (PBS). After 24 h, cells were examined under a microscope (Leica Microsystems, Wetzlar, Germany) using an SA‐Gal working solution (pH 6.0) (Cell Biolabs, CA, USA).

### 2.7 UVA/UVB irradiation and TNF‐α treatment

HDFs were plated in 24‐well plates at a density of 2 × 10^4^ cells/well per well and incubated for 24 h. Cells were exposed to UV irradiation (Boteck, Gunpo, Republic of Korea), specifically, 10 mJ/cm^2^ UVA and 40 mJ/cm^2^ UVB, after rinsing twice with PBS. Following UV exposure, cells were cultured in a medium with or without 0, 100, 200, 500, and 1000 ng/mL APNVs for a further 24 h.

HDFs were stimulated with tumour necrosis factor‐alpha (TNF‐α) at a concentration of 10 ng/mL, followed by treatment with 0, 100, 200, 500, and 1000 ng/mL APNVs for 24 h at 37°C in a humidified environment containing 5% CO_2_.

### 2.8 MMP‐1 and type I procollagen assay

Supernatant fractions and culture media from HDFs were used for experiments. According to the manufacturer's instructions, MMP‐1 and type I procollagen production measurements were performed using Procollagen Type I C‐Peptide EIA (Takara Bio, Kusatsu, Japan) and human MMP‐1 ELISA kits from R&D Systems (Minneapolis, USA).

### 2.9 Human skin equivalent preparation

The human skin equivalent model, Neoderm‐ED, was purchased from TEGO Science (Seoul, Korea) (Park et al., 2021). In brief, HDFs were seeded in the collagen matrix for 1 day. Next, keratinocytes were seeded on top of the collagen matrix and co‐cultured for 4 days. Following the removal of keratinocytes, the human dermal fibroblast block was exposed to air. We investigated three different conditions: irradiation with 10 mJ/cm^2^ UVA three times at 4‐h intervals, 40 mJ/cm^2^ UVB three times at 4‐h intervals, and treatment with 10 ng/mL of TNF‐α. Then skin models were treated with 1000 ng/mL of APNVs or EGCG, respectively. The human skin equivalent was incubated with the samples for 72 h at 37°C in an atmosphere of 5% CO_2_.

### 2.10 Immunohistochemical (IHC) analysis

Human skin equivalent samples were fixed in 4% paraformaldehyde and staining was performed by Tego Science (Seoul, Korea). Paraffin‐embedded, fixed skin tissues were sectioned at a thickness of 3 µm, deparaffinized with xylene, and rehydrated in a decreasing alcohol series with distilled water. Tissue sections were stained with hematoxylin and eosin (H&E) and Masson's trichrome. Histological changes were examined and images were obtained under a microscope (Leica Microsystems, Wetzlar, Germany).

### 2.11 RNA extraction and NanoString nCounter assay

Total RNA was extracted from cells using the MasterPure Complete DNA/RNA Purification Kit (Lucigen‐Epicentre^,^ Madison, USA) according to the manufacturer's instructions. A DS 11 Spectrophotometer (Denovix Inc., Wilmington, DE, USA) was used to measure the concentrations of extracted RNA for samples with > 100 ng total RNA. Additionally, a Fragment Analyzer (Advanced Analytical Technologies, Oak Tree Ct, Ankeny, IA, USA) was employed to assess the purity of RNA. For analysis of mRNA expression, the NanoString nCounter assay (Nanostring Technologies, Seattle, WA, USA) was used. Except for the DNA tags ligated to the 3′ end of each mRNA in samples, all tags were removed. After the identification of unique miRNAs, samples were hybridized with nCounter capture and barcoded DNA‐tag reporter probes. Using a 5× diluted sample preparation reaction solution (5 mL), hybridization reactions were carried out. Total RNA samples (100 ng) were incubated at 64°C for a minimum of 18 h. Subsequently, all excess capture and reported probes were removed. To categorize target RNA molecules within samples, each fluorescent barcode was counted using the nCounter digital analyzer.

### 2.12 Module analysis of the protein–protein interaction (PPI) network

Cytoscape was employed to visualize gene interactions in colour and significant genes according to node size, expressed as fold changes of differential expression (DE). PPI networks were analyzed using molecular complex identification, a novel graphic theoretic clustering algorithm that detects densely connected regions within protein networks. The parameters in MCODE were set as follows: degree cutoff of 2, node score cutoff of 0.2, k‐core of 2, and maximum depth of 100.

### 2.13 Gene ontology (GO) and Kyoto Encyclopedia of Genes and Genomes (KEGG) pathway analyses

Using the GO enrichment analysis tool, biological processes, cellular components, and molecular function groups were explored. Additionally, KEGG pathway enrichment was conducted using the KEGG database as the reference to identify significant pathways of differentially expressed genes (with *p*‐values 0.05 and |gene fold changes| > 2).

### 2.14 Statistical analysis

All data are expressed as mean ± SEM of triplicate experiments. Statistical analysis was performed using one‐way analysis of variance (ANOVA) and Dunnett's test (GraphPad Prism Software Inc., San Diego, CA, USA). Differences were considered statistically significant at *p* < 0.05.

## 3 RESULTS

### 3.1 Characterization of APNVs

**FIGURE 1:**
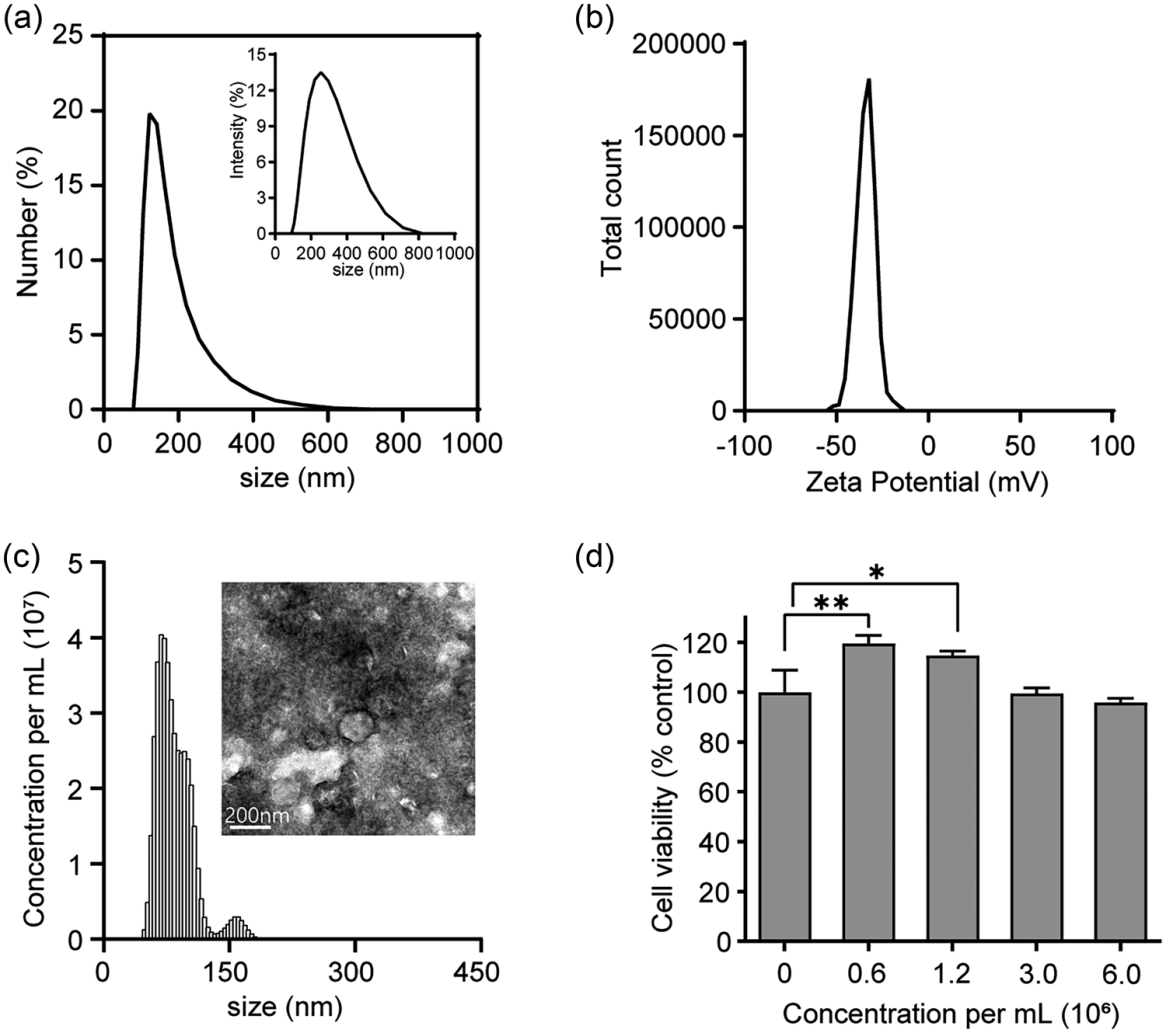
Characterization of APNVs. DLS measurements of particle size based on (a) intensity and number of APNVs. (b) Zeta potential distribution of APNVs. (c) NTA measurements of the APNVs concentration and TEM images of APNVs. Scale bar: 200 nm. (d) Viability of HDFs following treatment with APNVs. APNV, nanovesicles of *Artemisia princeps*; DLS, dynamic light scattering; NTA, Nanoparticle tracking analysis; TEM, transmission electron microscopy.

APNVs were purified via sequential centrifugation and filtration based on mammalian exosome separation procedures. Size distribution was assessed via dynamic light scattering (DLS) and nanoparticle tracking (NTA) analyses (Figure 1a–c). TEM images of the vesicles revealed an approximately spherical shape (Figure 1c), with estimated sizes ranging from 100 to 200 nm as determined from NTA and DLS measurements. The size of APNVs (30–200 nm) was comparable to that of naturally occurring mammalian exosomes. The APNVs concentration was 1.75 × 10^9^ particles/mL (Figure 1c) and the zeta potential was −35 mV (Figure 1b). Zeta potential provides an indication of dispersion stability, an attribute of the exosome population that reflects interactions between charged particles. Particles with higher zeta potential are more electrostatically mobile and have a stronger tendency to avoid aggregation. Vesicles with a minimal zeta potential of ± 20 mV or less are considered relatively stable. APNVs exhibited mutual repulsion and could potentially establish a stable suspension due to their slightly negative charge (35 mV) (Figure 1b). To evaluate the biological activity of APNVs, in vitro toxicity experiments were conducted for the determination of cell viability. According to data from cellular‐level experiments, APNVs at a concentration of 1000 ng/mL did not exert cytotoxic effects against HDFs after a 24 h treatment period (Figure 1d). Since the purpose of this study is to find the most effective concentration as a cosmetic ingredient, we chose 1000 ng/mL because it is the highest concentration amongst the safe concentrations in the cell viability experiment.

To further investigate the proliferative activity of HDFs for potential anti‐ageing effects, we treated HDF with APNV at concentrations ranging from 100 to 1000 ng/mL. We observed that the relatively low concentrations of 100–200 ng/mL induced peak proliferation rates more rapidly compared to the higher concentrations of 500–1000 ng/mL. Additionally, at all tested concentrations, HDF proliferation increased up to 9 days post‐incubation, plateaued or decreased by day 12, and then reached the highest proliferation rate at day 15, followed by a sharp decline (Figure S1).

### 3.2 Anti‐aging effect of APNVs in the passage‐associated senescence model

**FIGURE 2:**
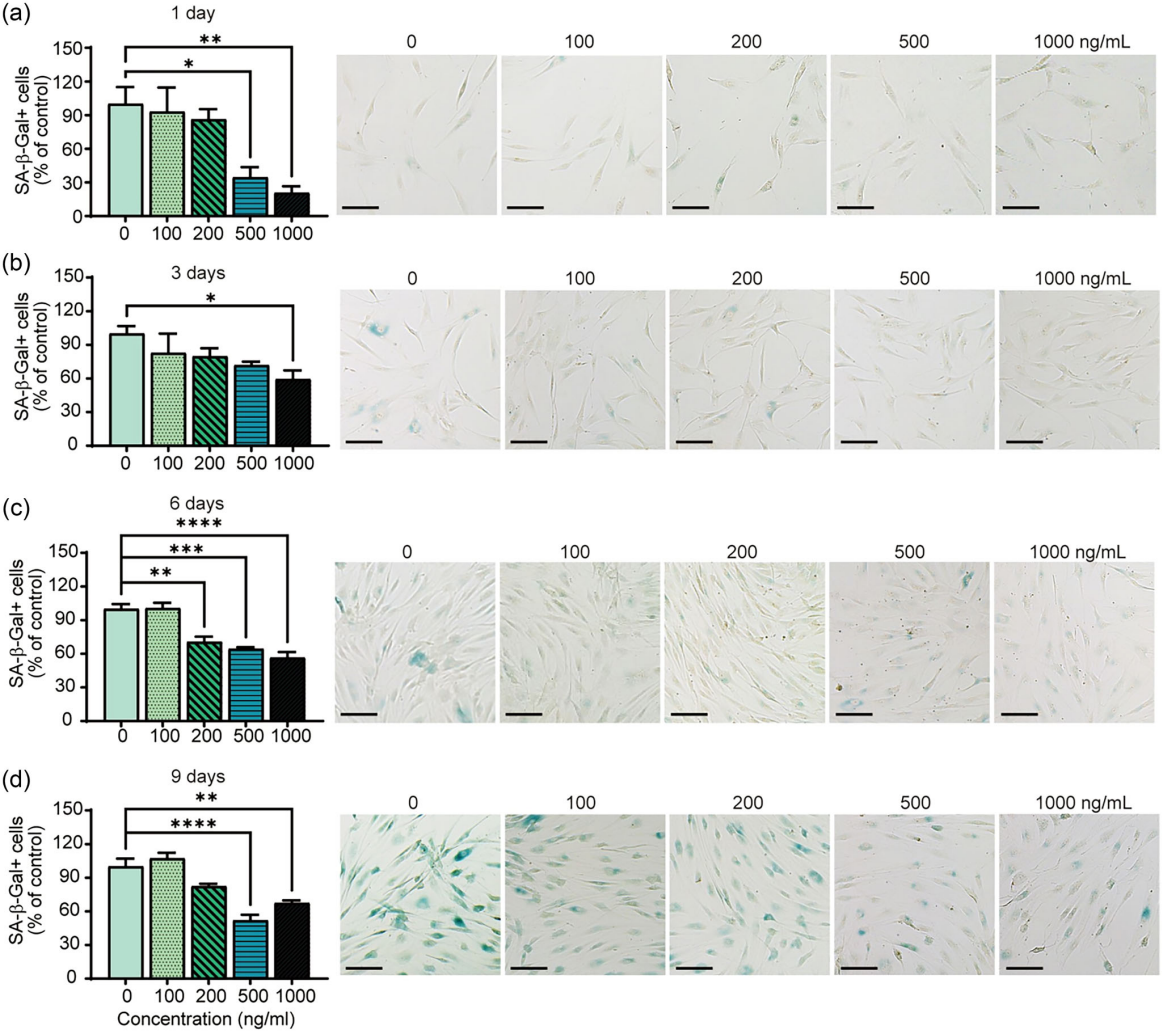
Effects of APNV treatment on the passage‐associated senescence model. Percentage of β‐gal positive cells and images showing positive β‐gal staining indicative of ageing cells at (a) 1 day, (b) 3 days, (c) 6 days, and (d) 9 days. APNV, nanovesicles of *Artemisia princeps*.

To evaluate the anti‐ageing properties of APNVs, we treated a passage‐related ageing model with APNVs and examined cell proliferation (Figure 2a‐d) (Kim et al., 2021; Shlush et al., 2011). We applied the SA‐β‐galactosidase experiment, known as a biomarker of cellular ageing (Wang & Dreesen, 2018), to determine whether APNVs induce a decrease in β‐galactosidase activity in aged HDFs. Quantitative data from the SA‐β‐galactosidase assay show that APNVs reduce the proportion of senescent cells, supporting the notion that APNVs suppress the level of senescence in HDFs (Figure 2).

SA‐β‐galactosidase activity decreased in a concentration‐dependent manner from 1 to 6 days after culture (Figure 2a–c), with the lowest activity observed at 500 ng/mL on day 9 (Figure 2d). Our findings provide strong evidence that APNVs reduce ageing in a concentration‐dependent manner. In particular, at a concentration of 1000 ng/mL, it was confirmed that the activity continued to decrease compared to the control group, even though cell proliferation continued from day 1 to day 9 after culture. Since the purpose of this study is to find the most effective concentration for use as a cosmetic ingredient, we selected 1000 ng/mL for additional experiments. This concentration is the highest amongst safe concentrations in cell viability experiments and consistently showed significant results in ageing models.

### 3.3 Effects of APNVs on MMP‐1 inhibition and type I pro‐collagen synthesis

**FIGURE 3:**
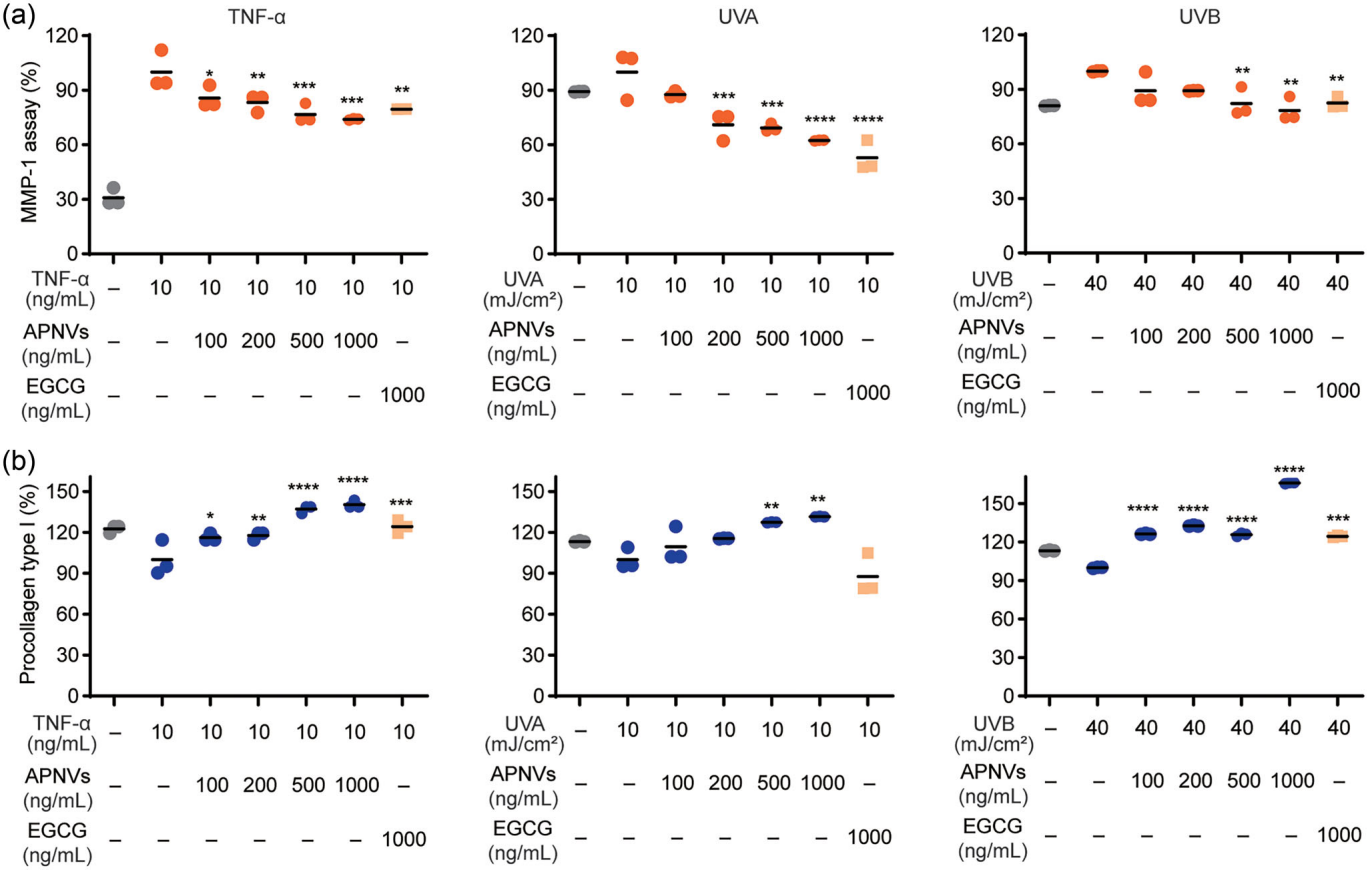
Effects of APNVs on levels of MMP‐1 and procollagen type I in HDFs. (a) MMP‐1 expression of HDFs in response to treatment with APNVs. (b) Procollagen expression of HDFs following treatment with APNVs. Cells were pretreated with TNF‐α or irradiated with UVA or UVB. Data are presented as mean ± standard error of the mean (SEM) (*p < 0.05, **p < 0.01, ***p < 0.001, ****p < 0.0001). APNV, nanovesicles of *Artemisia princeps*; HDFs, human dermal fibroblasts; MMP‐1, matrix metalloproteinase‐1; TNF‐α, tumour necrosis factor alpha.

Collagen is specifically denatured by MMPs that are directly responsible for the breakdown of ECM components. UV irradiation triggers increased synthesis of MMPs in the skin, in turn, causing the breakdown of ECM (Ågren et al., 2015; Cruz et al., 2023; Jung et al., 2014). Using (−)‐epigallocatechin‐3‐*O*‐gallate (EGCG) as a positive control, the impact of APNVs on MMP‐1 expression in HDFs after TNF‐α treatment and UVB exposure were examined (Figure 3) (Antonopoulou et al., 2016). MMP‐1 concentrations were assessed via ELISA after treatment of cells with 0, 100, 200, 500, and 1000 ng/mL of APNVs for 24 h. In cells exposed to TNF‐α, UVA, and UVB, APNVs induced a decrease in MMP‐1 expression. In particular, treatment with 1000 ng/mL APNVs led to a significant reduction of MMP‐1 expression (by 26% in the TNF‐α group, 37.6% in the UVA group, and 21.6% in the UVB group; Figure 3a). Moreover, APNV‐mediated suppression of MMP‐1 was dose‐dependent. Recent studies have demonstrated that UV radiation promotes collagen breakdown and inhibits the synthesis of type I procollagen in fibroblasts (Figure 3b) (Lee et al., 2014; Lee et al., 2019). In the current study, extracts of APNVs induced a significant increase in type I procollagen synthesis (with restoration of 40% in TNF‐α, 31.5% in UVA, and 66% in UVB treatment groups, respectively, at a concentration of 1000 ng/mL). Under UVB exposure, procollagen synthesis by HDFs treated with APNVs was significantly higher relative to control and EGCG treatment groups. Overall, APNVs suppressed the expression of TNF‐α, UV‐induced MMP‐1 and increased type I collagen protein in HDFs.

### 3.4 Significant differentially expressed genes in APNV‐treated HDFs exposed to TNF‐α, UVA, and UVB

**FIGURE 4:**
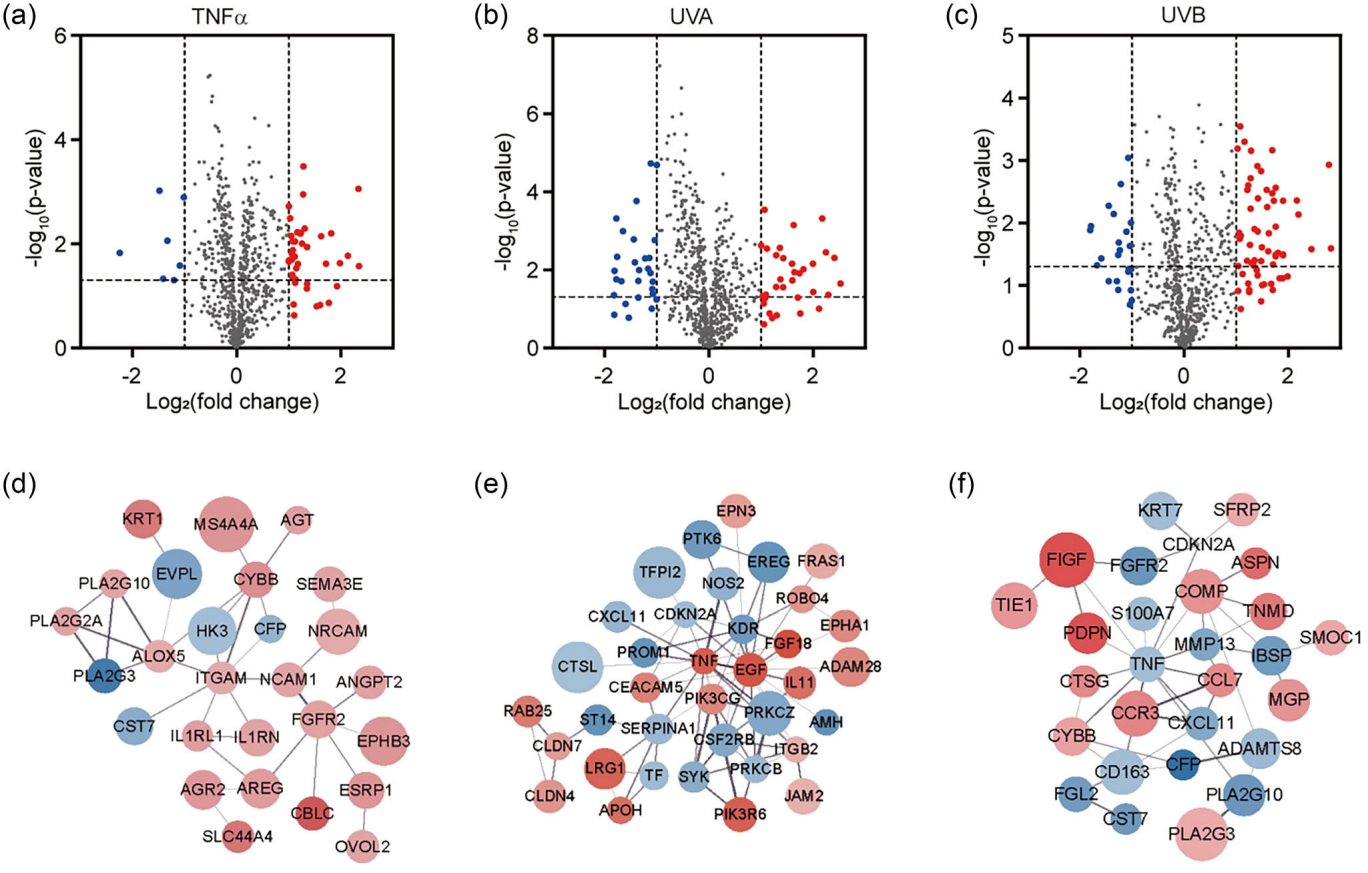
Identification of differentially expressed genes in TNF‐α, UVA, and UVB‐exposed cell groups following treatment with APNVs. Volcano plot showing differences in gene expression in (a) TNF‐α, (b) UVA, and (c) UVB‐treated groups upon incubation with APNVs. Top module of the protein–protein interaction (PPI) network for densely connected nodes of (d) TNF‐α, (e) UVA, and (f) UVB groups with APNV treatment. Red, DE genes with log2 (fold change) > 1; blue, DE genes with log2 (fold change) < −1. A larger node size signifies a more significant *p*‐value. APNV, nanovesicles of *Artemisia princeps*; TNF‐α, tumour necrosis factor alpha.

**FIGURE 5:**
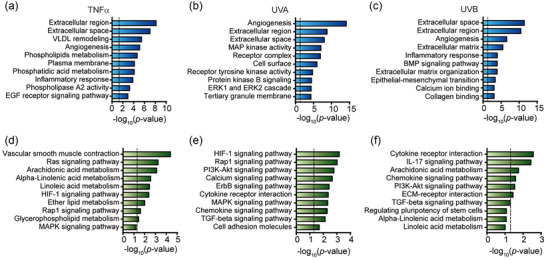
Expression profiling in TNF‐α, UVA, and UVB‐exposed cell groups following treatment with APNVs. GO term enrichment analysis of expressed mRNAs in (a) TNF‐α, (b) UVA, and (c) UVB treatment groups upon incubation with APNV. KEGG pathway analysis of differentially expressed mRNAs in (d) TNF‐α, (e) UVA, and (f) UVB treatment groups upon incubation with APNVs (*p*‐value < 0.05, |log2 (fold change)| > 1). The dashed line signifies a *p*‐value of 0.05. APNV, nanovesicles of *Artemisia princeps*; GO, Gene ontology; KEGG, Kyoto Encyclopedia of Genes and Genomes; TNF‐α, tumour necrosis factor alpha.

To identify the genes in HDFs from TNF‐α and UV treatment groups affected by APNVs, mRNA expression was profiled using the Nanostring nCounter platform. To this end, HDFs exposed to TNF‐α and UV were treated with 1000 ng/mL APNVs and differences in gene expression were assessed. DEGs showing marked changes (determined as |log2 (fold change)| > 1) following APNVs treatment, compared with gene levels in HDFs exposed to TNF‐α and UV (*p*‐value < 0.05) were selected. The profiles of selected DEGs were visualized as volcano plots (Figure 4a–c). Overall, ∼0.9%–3.9% of genes were downregulated and 4.3%–7.9% of genes were upregulated in TNF‐α‐ and UV‐exposed HDFs treated with APNVs (Figure 4). Next, we performed GO enrichment analysis of differentially expressed mRNAs with critical functions, comparing HDFs treated with TNF‐α and UV in the absence and presence of APNVs, as shown in Figure 5. The top 10 differently expressed mRNAs were identified via GO enrichment and KEGG pathway analyses. Analysis of the enriched GO terms within DEGs following APNVs treatment revealed significant enrichment of extracellular matrix (ECM)‐related functions (Figure 5). For TNF‐α‐treated HDFs incubated with APNVs, the most enriched GO terms were phosphatidylethanolamine metabolic process (*p*‐value < 0.0001), phosphatidic acid metabolic process (*p*‐value < 0.0001), inflammatory response (*p*‐value < 0.0001), and calcium‐dependent phospholipase A2 activity (*p*‐value < 0.0001), as shown in Figure 5a. For UVA‐exposed HDFs incubated with APNVs, the most enriched GO terms were angiogenesis (*p*‐value < 0.0001), receptor complex (*p*‐value < 0.0001), cell surface (*p*‐value < 0.0001), and transmembrane receptor protein tyrosine kinase activity (*p*‐value < 0.0001), as shown in Figure 5b. For UVB‐exposed HDFs incubated with APNVs, the most enriched GO terms were inflammatory response (*p*‐value < 0.0001), ECM organization (*p*‐value < 0.0001), and collagen binding (*p*‐value < 0.0001), as shown in Figure 5c. The collective results indicate that genes related to the ECM and cell membrane were amongst those most significantly affected by APNV treatment of HDFs exposed to TNF‐α and UV. Data from the KEGG pathway enrichment analysis were consistent with those of the GO enrichment analysis (Figure 5d–f). For example, following APNV treatment, pathways such as arachidonic acid metabolism in TNF‐α‐treated HDFs, HIF‐1 signalling in UVA‐exposed HDFs, and cytokine‐cytokine receptor interactions in UVB‐exposed HDFs were also enriched (Figure 5).

### 3.5 Effects of APNVs on histological changes in a 3D skin model

**FIGURE 6:**
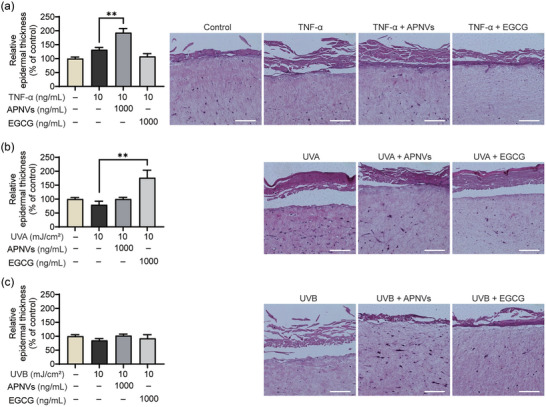
Effects of APNVs on histological changes in a human skin equivalent model. Representative H&E‐stained images and epidermal thickness of H&E‐stained sections were evaluated via microscopy for the (a) TNF‐α, (b) UVA, and (c) UVB treatment groups. Data are presented as mean ± standard error of the mean (SEM) (*p < 0.05, ***p < 0.001). APNV, nanovesicles of *Artemisia princeps*; H&E, hematoxylin and eosin; TNF‐α, tumour necrosis factor alpha.

**FIGURE 7:**
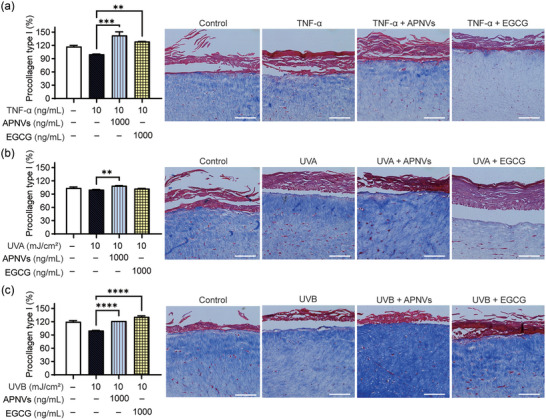
Effects of APNVs on collagen expression in a human skin equivalent model. Masson's trichrome staining for visualization of collagen fibre for the (a) TNF‐α, (b) UVA, and (c) UVB treatment groups. Data are presented as mean ± SEM (**p < 0.01, ***p < 0.001, ****p < 0.0001). APNV, nanovesicles of *Artemisia princeps*; TNF‐α, tumour necrosis factor alpha; SEM, standard error of the mean.

In an in vitro assay using ECM of the dermis, APNVs improved collagen function related to skin elasticity and hydration. We treated a reconstructed 3D human skin model with TNF‐α and UVs to induce histological changes, including destruction of the epidermis and degradation of collagen (Figure 6) (Son et al., 2020). Epidermal thickness is a characteristic used to evaluate aging of skin (Pageon et al., 2019). The effects of APNVs on TNF‐α‐, UVA‐, and UVB‐exposed human skin equivalent were investigated via histochemical analysis. The collagen content and thickness of the epidermal layer were initially evaluated. Notably, thickness of the epidermis of the TNF‐α‐treated 3D cultured artificial skin was considerably recovered (up to 46.4%) following incubation with 1000 ng/mL APNVs (Figure 6a). Additionally, Masson's trichrome staining was employed to determine collagen intensity (Figure 7) (Huh et al., 2015; Kang et al., 2020). Collagen fibers of the UVB‐irradiated skin equivalent were less dense and more erratically arranged relative to the dense, regular fibers of nonirradiated skin. Additionally, the groups that had been treated with APNVs showed significant accumulation of collagen in the dermis. In line with the in vitro results, the collagen content in the UVB‐irradiated human skin equivalent was restored (Figure 7c). These findings imply that APNVs effectively block the TNF‐α and UV‐induced alterations in epidermal thickness and collagen degradation. The ability of APNVs to modulate procollagen synthesis levels was further assessed using ELISA. As shown in Figure 7(a,c), treatment with APNVs significantly promoted procollagen synthesis.

## 4 DISCUSSION

*A. princeps* contains NVs that exhibit significant anti‐ageing effects on skin fibroblasts in vitro. This study investigated the impact of *A. princeps* NVs on collagen expression and anti‐ageing effects using reconstructed human keratinocyte and skin fibroblast models. Artemisia species are widely used for treating various diseases, such as malaria, hepatitis, cancer, tumours, and neuritis, as well as alleviating fever, swelling, and wound infections. Traditionally, these plants are used directly in folk medicine or indirectly as pharmaceutical agents in modern medicine. The increasing global interest in plant research is supported by substantial evidence of the immense potential of medicinal plants in various traditional systems. Medicinal plants are primary sources of bioactive compounds with therapeutic value (Csekes & Račková, 2021). NVs, excluding secondary metabolites generated for defence, have recently gained attention as plant‐derived materials (Nemati et al., 2022; Suharta et al., 2021).

Previous studies have shown that leaf‐derived extracellular vesicles (LEVs) exhibit anti‐melanogenic effects, while sap‐derived EVs display anti‐metastatic and cytotoxic effects on various tumour cell types (Kim, Yoo et al., 2020; Kim, Jung et al., 2020). Plant‐derived extracellular vesicles (PDVs) contain DNA, mRNA, miRNA, proteins, lipids, and compounds with protective functions, generated in response to various biotic and abiotic stress factors, including pathogen infections (Chen et al., 2021; Shinge et al., 2022). These PDVs are being recognized as natural drug delivery nanoplatforms and therapeutic agents (Hajialyani et al., 2018; Zhuang et al., 2015). The APNVs in this study were found to have beneficial effects on aged skin. To our knowledge, this is the first study to investigate the anti‐ageing effects of *A. princeps* NVs using human skin models. This study enhances the understanding of APNVs' role in ageing skin and establishes a scientific basis for their therapeutic activity.

GO enrichment analysis revealed that genes involved in the phosphatidylethanolamine metabolic process and phosphatidic acid metabolism are implicated in the effects of APNVs on TNF‐α. The lipid composition of cell membranes is crucial in the ageing process. The pro‐inflammatory cytokine TNF‐α accumulates with age and is associated with age‐related diseases (Csekes & Račková, 2021; Rea et al., 2018). Changes in the lipid content of cell membranes affect both cellular function and ageing (Das, 2021). TNF‐α activation stimulates phospholipase A2, leading to the release of arachidonic acid, which can integrate into inflammation‐related signalling networks (Stromsnes et al., 2021; Yang et al., 2014).

Solar ultraviolet radiation (UVR) is one of the most potent natural physical carcinogens associated with photoaging (Deng et al., 2018) and a major factor in chronic inflammation and skin ageing. GO enrichment analysis indicated that genes upregulated in response to UVA and APNV treatment are involved in angiogenesis and related to hypoxia‐inducible factor‐1 (HIF‐1) signalling. UV responses are linked to HIF‐1α activation and hypoxia signalling, which can regulate angiogenesis. This theory is further supported by findings that HIF‐1 target genes, such as heme oxygenase‐1 (HO‐1), are regulated in the skin by UV (Wunderlich et al., 2008).

HIF‐1α is a stress protein responsive to both hypoxia and excessive oxygen (Cho et al., 2012). Recent studies have elucidated the role of HIF‐1α in UVR response as a transcription factor that can balance inflammatory signals or cell apoptosis (Wei et al., 2022). Proper regulation of HIF in cells exposed to UVA after APNV treatment may help suppress inflammatory responses post‐disease or stress. GO enrichment data indicated that APNV treatment affected inflammation and ECM tissue pathways in UVB‐exposed cells. Tumour necrosis factor (TNF) and MMP13 genes related to inflammatory responses decreased in volcano plots. UV exposure initiates ageing‐related cascades, inducing MMP‐mediated and inflammation‐induced ageing processes responsible for ECM degradation (Cho et al., 2022; Kim et al., 2016; Lago & Puzzi, 2019). APNV treatment significantly reduced the expression of genes associated with inflammatory factors, suggesting substantial anti‐inflammatory effects.

As inflammation and skin ageing are closely related, evaluating anti‐inflammatory effects can indirectly indicate anti‐ageing therapeutic efficacy (Cai et al., 2020; Frenk & Houseley, 2018). Modulation of inflammatory phases can reduce inflammatory responses and ageing processes through the regulation of arachidonic acid metabolism, HIF‐1 signalling, and chemokine signalling pathways.

Medicinal plants are considered for numerous applications due to their biological efficacy and safety. An increasing number of plant compounds are being identified as potential anti‐ageing agents. Thus, a comprehensive understanding of the signalling mechanisms underlying gene regulatory patterns can foster the development of anti‐ageing therapeutic strategies and lead to new clinical targets and treatment strategies.

This study focused on the anti‐ageing effects of APNVs using human skin fibroblasts and 3D skin models. We clearly observed the dose‐dependent effects of APNVs on MMP‐1 inhibition and type I procollagen synthesis and identified key genes related to TNF‐α and UV‐induced effects influenced by APNVs through genotyping analysis. Results obtained using 3D in vitro models demonstrated the potential of these naturally occurring plant‐derived nanoscale molecules to exert inhibitory effects on stress responses. Genes with DE patterns in response to APNVs were primarily associated with ECM‐related functions and inflammatory responses. Based on the identification of APNVs as novel bioactive carriers with anti‐ageing properties, we propose that an integrated nanomedicine approach using natural plant‐based nanotechnology could offer innovative directions for improving targeted substrate therapy.

## AUTHOR CONTRIBUTIONS

**Ju Hun Yeon**: Conceptualization. **Ju Hun Yeon and Kimin Kim**: Data curation. **Kimin Kim and Yehjoo Sohn**: Formal analysis. **Ju Hun Yeon**: Funding acquisition. **Kimin Kim and Yehjoo Sohn**: Investigation. **Ju Hun Yeon**: Methodology. **Ju Hun Yeon**: Project administration. **Ju Hun Yeon**: Resources. **Ju Hun Yeon**: Supervision. **Kimin Kim**: Validation. **Kimin Kim and Yehjoo Sohn**: Visualization. **Kimin Kim**: Writing—original draft. **Ju Hun Yeon and Yehjoo Sohn**: Writing—review and editing. All authors have read and agreed to the published version of the manuscript.

## CONFLICT OF INTEREST STATEMENT

The authors have no conflicts of interest to declare.

## Supporting information



Supporting Information
