# Supplementary material for: Anti‐ageing activities of nanovesicles derived from Artemisia princeps in human dermal cells and human skin model
Source: J Extracell Biol. 2025 Apr 24;4(4):e70033. doi: 10.1002/jex2.70033 (PMC12021671; doi:10.1002/jex2.70033)
Supplement: Supplementary file 1 — Supporting Information [file JEX2-4-e70033-s001.docx]

**Supplementary Data**

**Anti-aging activities of nanovesicles derived from *Artemisia princeps* in human dermal cells and human skin model**

Kimin Kim^1^, Yehjoo Sohn^1^, Ju Hun Yeon^1,2*^

^1^Department of Integrative Biosciences, University of Brain Education, Cheonan, Republic of Korea

^2^Well-aging Exobio Inc., Cheonan, Korea

*Correspondence: Ju Hun Yeon

Department of Integrative Biosciences, University of Brain Education, Cheonan, Republic of Korea

Tel +82 41 529 2621

Fax +82 41 529 2674

E-mail jhyeon@ube.ac.kr


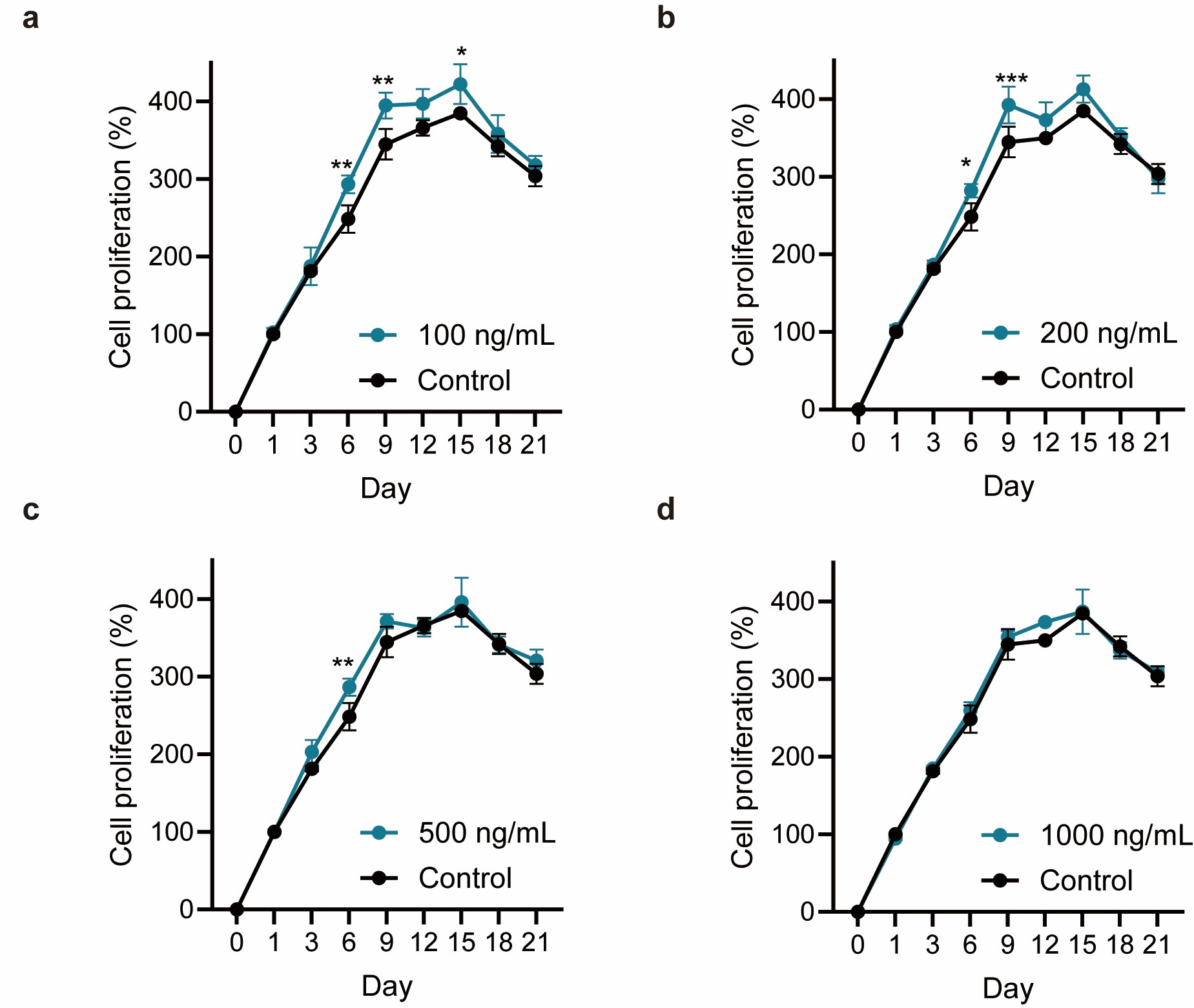


**Fig. S1. Proliferation effects of nanovesicles from *Artemisia princeps* (APNVs) on human dermal fibroblast cells.** APNVs were treated to senescent fibroblast cell with passage 10 over, and cultured for 21 days. Cell proliferation of HDFs following treatment with APNVs for (a) 100, (b) 200 ng/mL, (c) 500, and (d) 1000 ng/mL. Data are presented as means ± standard error of mean (SEM) (**p* < 0.05, ***p* < 0.01, ****p* < 0.001).
